# A realistic fabrication and design concept for quantum gates based on single emitters integrated in plasmonic-dielectric waveguide structures

**DOI:** 10.1038/srep28877

**Published:** 2016-07-01

**Authors:** Günter Kewes, Max Schoengen, Oliver Neitzke, Pietro Lombardi, Rolf-Simon Schönfeld, Giacomo Mazzamuto, Andreas W. Schell, Jürgen Probst, Janik Wolters, Bernd Löchel, Costanza Toninelli, Oliver Benson

**Affiliations:** 1 AG Nano-Optik, Institut für Physik, Humboldt-Universität zu Berlin, Newtonstraβe 15, 12489 Berlin, Germany; 2 Helmholtz-Zentrum Berlin für Materialien und Energie, Albert-Einstein-Straβe 15, 12489 Berlin, Germany; 3 European Laboratory for Non-Linear Spectroscopy (LENS), University of Florence, Via Nello Carrara 1, 50019 Sesto Fiorentino Florence, Italy; 4 INO, Istituto Nazionale di Ottica, Largo Fermi 6, 50125 Firenze, Italy; 5 Department of Electronic Science and Engineering, Kyoto University, Kyoto daigaku-katsura, Nishikyo-ku, 615-8510 Kyoto, Japan; 6 Departement Physik, Universität Basel, Klingelbergstrasse 82, CH-4056 Basel, Switzerland

## Abstract

Tremendous enhancement of light-matter interaction in plasmonic-dielectric hybrid devices allows for non-linearities at the level of single emitters and few photons, such as single photon transistors. However, constructing integrated components for such devices is technologically extremely challenging. We tackle this task by lithographically fabricating an on-chip plasmonic waveguide-structure connected to far-field in- and out-coupling ports via low-loss dielectric waveguides. We precisely describe our lithographic approach and characterize the fabricated integrated chip. We find excellent agreement with rigorous numerical simulations. Based on these findings we perform a numerical optimization and calculate concrete numbers for a plasmonic single-photon transistor.

Semiconductor transistors are based on non-linear responses that enable signal control by means of a weak gating signal^1^. While in electronics strong Coulomb interaction allowed continuous improvement leading to extremely efficient transistors working with low switching currents, non-linear optical devices suffer from very low non-linear coefficients and thus usually require high-intensity laser beams. Nevertheless, in recent years promising concepts have been proposed and realized^2^,^3^,^4^,^5^,^6^ operating even at the few to single photon level by exploiting individual quantum systems as non-linear elements. In order to achieve the desired non-linear response - ideally in an integrated device - a sufficiently strong interaction between single emitters and confined light is mandatory. The interaction strength can be enhanced by the use of emitters with a large transition dipole moment, or by resonantly enhancing and tightly confining the electromagnetic field. Dielectric micro-resonators^7^,^8^,^9^,^10^ and plasmonic nano-resonators^11^,^12^ have been used for this purpose.

Recently, more broadband solutions, where emitters interact with freely propagating (focused) light^5^ or guided modes have gained attention^13^,^14^,^15^,^16^. Especially the use of guided surface plasmon polaritons (SPPs) promises a small footprint and huge Purcell enhancements. In particular, guided SPPs feature the advantage that high field intensities are found outside of the waveguides, i.e., they have an intense near-field. In contrast, the highest field intensities of dielectric waveguides are typically confined inside the material. Additionally, plasmonic waveguides have the advantage that the guided energy density can be significantly increased by shrinking the waveguide dimension. Especially, the fundamental mode does not suffer from an increasing spread of the evanescent part into the surrounding but the confinement increases^14^. Thus, the energy density of a propagating SPP can be further increased compared to purely photonic modes (resulting in higher non-linearities) and the corresponding fields are accessible from the outside^17^ rendering SPPs interesting for functionalization with single emitters in *hybrid* approaches. In contrast to approaches that use directly incorporated emitters^18^, assembly of hybrid devices allows for independent optimization of the quantum emitters and the light guiding structures^19^. Accordingly, theoretical proposals predict switches working at the single SPP level^4^ and devices achieving strong coupling of emitters to propagating SPPs^14^.

We show here how the rather idealized system of a single emitter controlling the transmission of SPPs in a cylindrical nanowire considered in the proposal by Chang *et al*.^4^ can be actually transformed into a practical on-chip device. First, we introduce an elaborate fabrication process allowing for the realization of highly integrated and efficient plasmonic-dielectric elements. The excellent agreement between the experimental optical response and the corresponding numerical simulation then enables us to provide a realistic design for an integrated plasmonic single-photon transistor. In particular, we investigate a structure that is optimized for single molecules as sources of the non-linearity. Concrete parameters for experimentally expectable non-linearities and throughput are provided.

## Results

### Design considerations for a quantum plasmonic non-linear element

For an experimental realization of a single photon non-linear device, based on a hybrid approach like discussed before, there are two key ingredients:

- An efficient, integrated dielectric-plasmonic structure that allows for high coupling rates between emitter and guided mode, i.e., high Purcell factors and low quenching rates,
- A stable single quantum emitter with predictable properties, large optical dipole moment, and – importantly – with a large branching ratio *η*_ZPL_ of emission into its zero phonon line (ZPL) with respect to phonon-assisted emission (*η*_ZPL_ is also referred to as (photonic) Debye-Waller factor).

In this paper, we focus on the dielectric-plasmonic structure. However, with respect to emitters we refer to organic molecules throughout this paper. Even more specific we consider dibenzoterrylene (DBT) hosted in anthracene (AC) crystals with emission lines around 785 nm. This bright emitter features high photo-stability and a narrow lifetime-limited linewidth at cryogenic temperatures^20^ with a high branching ratio of up to *η*_ZPL_ = 50%^21^. Assembly of the hybrid system would be finally performed by spincoating of a DBT-toluene mixture with AC in diethylether or by evaporation of this solution onto the chip. By this, DBT-doped AC crystals will form all over the chip. Here the aim is to avoid the need of a deterministic placement of individual DBT molecules. When the linewidth of the emitters is decreased to its Fourier-limit (<100 MHz) by cooling in a cryostat (to ≈2 K), individual emission and absorption lines are selectively accessible due to the inhomogeneous broadening. In this way emitters can be addressed individually by bringing the excitation laser into resonance with a specific absorption line. Thus, a very high density of DBT emitters can be used, without losing the possibility to address individual emitters, which would be the case at room temperature. This means that thousands of molecules can be hosted even in small volumes like that of a laser focus. At such high densities, finding well-positioned emitters deposited in the near-field region of the plasmonic waveguide is most likely which circumvents the need for spatial post-alignment.

**Figure 1:**
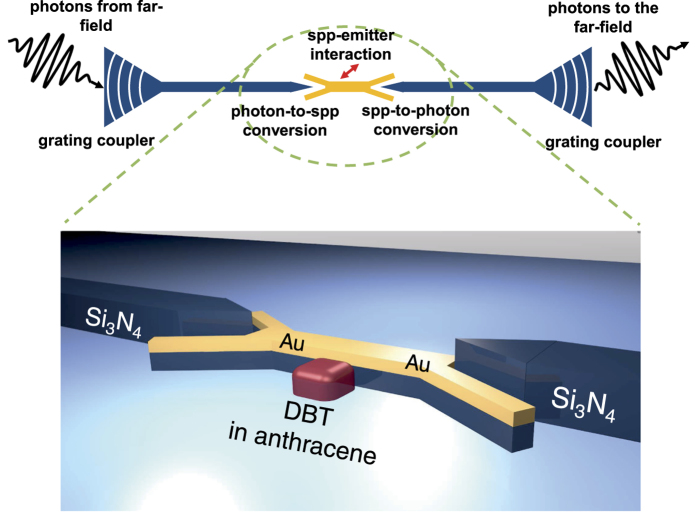
Scheme of a single photon non-linear device. A stream of photons is coupled via a dielectric grating coupler to a dielectric waveguide made from Si_3_N_4_ (blue color) which guides the photons to a V-shaped photon-to-plasmon transducer, where SPPs in a short plasmonic gold stripe waveguide (golden color) are excited. Well aligned emitters (e.g., DBT molecules in AC) experiencing high Purcell factors in the near-field of the plasmonic waveguide, intensely interact with the SPPs. After the interaction, SPPs are transduced back to photons and guided away by a second dielectric waveguide. These photons are eventually coupled out through a second grating coupler.

The efficiency of an integrated dielectric-plasmonic structure working on the single photon and single emitter level depends on efficient coupling of far-field optics with on-chip photonics and emitters. Especially SPPs generated on the chip must be extracted with high efficiency to allow for good signal-to-noise ratios in experiments. Further, on the chip, an efficient coupling to SPPs is needed while keeping propagation losses as small as possible. Coupling into (and out of) the integrated dielectric waveguides is here performed via Bragg-grating far-field couplers^22^. Moreover, an earlier optimized photon-to-plasmon transducer geometry (see ref. 23) allows for very high single mode conversion. Finally, the excited SPP is supposed to interact strongly with a closely positioned emitter. It is essential to address a plasmonic mode that provides a high Purcell factor *P* = Γ_WG_/Γ_0_ and a high (ideally near-unity) *β*-factor: *β* = Γ_WG_/Γ_tot_ (where Γ_tot_ is the emitter’s total decay rate, Γ_WG_ the coupling rate to the guided SPP and Γ_0_ is the vacuum decay rate of the emitter). Strong field enhancement close to the plasmon waveguide does not guarantee that the emitter’s decay rate Γ_tot_ is dominated by the emission into a single guided SPP mode with rate Γ_WG_^14^. In a realistic scenario various channels can contribute to Γ_tot_, such as coupling to weakly guided or radiating modes^24^, the presence of more than one bound mode in waveguides^25^ and especially quenching by surface energy transfer (SET)^26^. At the same time, the coupling strength to the plasmonic mode has to be balanced with the unavoidable propagation losses. The plasmonic waveguide has to be as short as possible without introducing direct coupling between dielectric waveguides which would undermine the signal-to-noise ratio of the entire device: ideally any photon that passes the integrated structure must have interacted with the emitter. The design must allow for practical coupling of emitters after fabrication. Figure 1 illustrates schematically our (symmetric) device architecture. A Bragg-grating coupler funnels far-field photons into a dielectric Si_3_N_4_ waveguide. Then, an efficient V-type photon-to-plasmon transducer (details of its design and numerical simulation via finite element method (FEM) in ref. 23) transfers excitations into a short SPP-waveguide with tight confinement. There, organic dibenzoterrylene (DBT) molecules precisely positioned next to the plasmonic waveguide will either reflect or give way to incoming single SPPs depending on the molecule’s internal state and the detuning between SPP and optical transition of the emitter, yielding a non-linear behavior. Finally, transmitted SPPs are converted into guided photons and eventually coupled into free-space again.

### Fabrication of integrated plasmonic-dielectric structures

**Figure 2:**
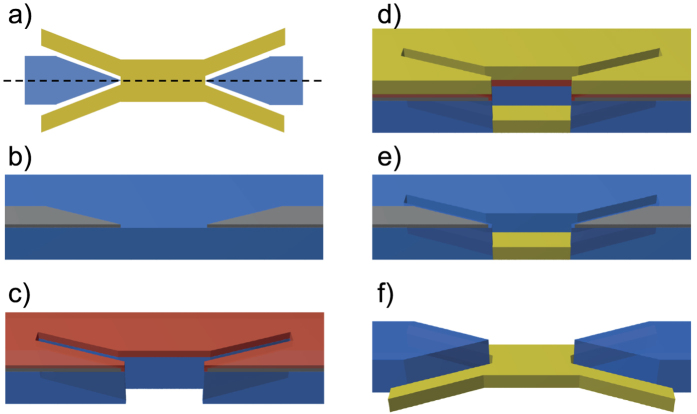
Simplified overview of the different steps during processing of the photonic-plasmonic on-chip structures. (**b–e**) Cut along the dashed line in (**a**). (**b**) Ni mask (grey) for the dielectric waveguides in Si_3_N_4_ (blue), (**c**) ZEP resist (red)/Si_3_N_4_ mask for the plasmonic components after the second etching step, (**d**) gold evaporation (yellow layer comprises Cu, Au and Ni layers), (**e**) lift-off of gold on ZEP resist, (**f**) final etching step (and removal of remaining protective Ni on gold and dielectric structures).

**Figure 3:**
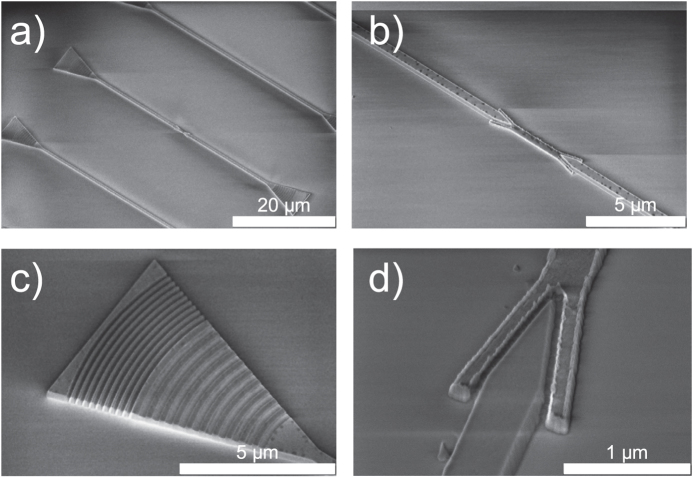
Scanning electron micrographs of the fabricated structures (**a–c**) before Ni removal). (**a**) Overview of the entire structure showing the dielectric grating couplers and the dielectric waveguides with a short plasmonic waveguide in its middle. The dielectric waveguides not containing a plasmonic waveguide above and below are used as references. (**b**) Zoom-in showing the plasmonic waveguide and the dielectric-plasmonic transducers. (**c**) The photonic grating-coupler, consisting of a partially etched grating and a Bragg-reflector. (**d**) Zoom-in to the photon-to-plasmon transducer with a 40 nm gap between metallic and dielectric components.

We fabricated an elaborate dielectric-plasmonic chip. The photonic part is built from a 300 nm layer of low pressure chemical vapor deposition (LPCVD) Si_3_N_4_, grown on a thermally oxidized silicon substrate (Silicon Valley Microelectronics, Inc.). Si_3_N_4_ is almost absorption-free in the desired wavelength range (785 nm). For the plasmonic part of the chip we use a bi-layer consisting of 5 nm Cu and 60 nm Au, evaporated on the chip. This material system represents a compromise between fabrication constraints, stability under ambient conditions and propagation losses by Ohmic resistance in the metallic structure. To produce the combined photonic-plasmonic structures two complementary lithographic techniques are used. The Si_3_N_4_ can only be structured by a subtractive method^27^, i.e., a positive etch-mask is fabricated and the nitride is removed via etching. In contrast, the plasmonic gold parts have to be built up via additive nanostructuring. Both methods need masks in opposite tones. In addition the additive structuring needs a sacrificial layer for a lift-off process. These circumstances and the gap between the dielectric and plasmonic part in the nanometer range impede a separation into consecutive structuring steps. Figure 2 gives an overview of the fabrication process of the transducer region. For the electron beam lithography (EBL) steps we use a Vistec EBPG5000plus system with 100 kV acceleration voltage, an Oxford plasmalab 80 plus for reactive ion etching (RIE) and a Leybold thermal evaporation system. In the first EBL step, square markers with an edge length of 20 *μ*m are fabricated using a Ti-Au-Ni-layer system (not shown in Fig. 2). Later on, these markers allow alignment of the three subsequent EBL steps for which an accuracy of at least 5 nm was found. In the second EBL step, the coupler gratings of the dielectric grating couplers (consisting of coupler grating and reflection grating) are fabricated (not shown in Fig. 2). A PMMA (2.2 M, Microchemicals) resist layer is used as an etch-mask and the structure is transferred to the substrate via a highly anisotropic CHF_3_-RIE-process (etch-depth 60 nm). In a third EBL step a nickel etch-mask for the dielectric waveguides and the reflecting gratings (Bragg mirrors) of the grating couplers are prepared. The recoated substrate (PMMA 2.2 M) is then exposed by EBL and the resulting positive mask is coated with 10 nm Ni by evaporation. The subsequent lift-off step is done using dimethylformamide (DMF) (Fig. 2b). To achieve the required small gaps between plasmonic and dielectric waveguide, the Si_3_N_4_-layer is also used as a mask for the plasmonic parts in the fourth lithography step. Therefore the substrate is coated with a 150 nm thick ZEP 7000 (Zeon Corporation, Japan) resist layer (burying the thin Ni etch-mask underneath it), which is structured with the positive tone of the plasmonic transducer. Compared to standard PMMA, the ZEP resist has a higher etch-resistance, thus the resist remaining after etching is still thick enough to be used as sacrificial layer in the following lift-off process. A thicker PMMA layer cannot be used as the high aspect ratio at the required structure sizes could not be achieved. The Si_3_N_4_ layer is partially removed in a highly anisotropic CHF_3_-RIE process at the designated positions of the plasmonic parts (Fig. 2c). At this step the control of the etch-depth allows adjusting of the vertical placement of the plasmonic transducer, which might be used in principle as an additional control parameter to achieve coupling efficiencies even higher than predicted in ref. 23. Actually, for the structures characterized in this study, the plasmonic parts are fabricated on top of a 115 nm Si_3_N_4_ plateau of the same cross section, elevating the plasmonic waveguide to the center of the dielectric waveguide (not shown in Fig. 2 for simplicity). This particular chip was used here, as its overall quality was very high. Adapted numeric simulations for this specific elevated design however reveal that its transducer efficiency is not optimal and will drop from 60% (in ref. 23) to around 40%. The plasmonic Cu-Au layer system is evaporated with a high working distance of 45 cm which results in only thin walls at the edges of the plasmonic transducer (Fig. 2d). The particular choice of Cu guarantees a smooth gold surface, Cu is not affected by the last wet etch-process and it has relatively small Ohmic losses. Additionally, the gold is covered with a 10 nm Nickel layer (not shown in Fig. 2 for simplicity) for high resistance to the final CHF_3_-RIE process. Removal of the sacrificial ZEP layer is again done with a DMF lift-off (Fig. 2e). The final process steps are the etching of the dielectric waveguides in a highly anisotropic CHF_3_-RIE process and the removal of the Ni etch-masks by wet etching with hydrochloric acid (Fig. 2f). Figure 3 shows electron micrographs of the fabricated structures before Ni removal.

### Characterization of fabricated structures

**Figure 4:**
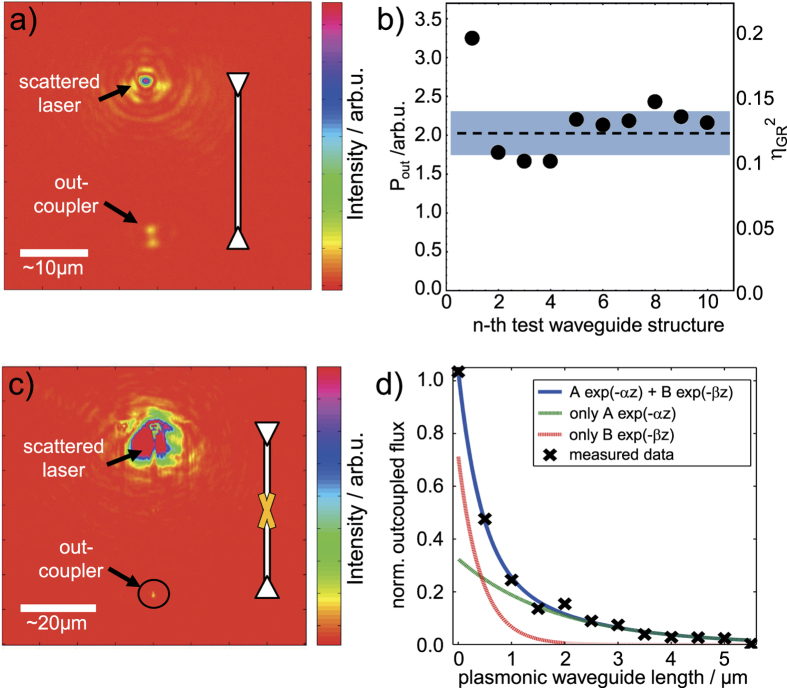
Experimental analysis of grating couplers (**a,b**) and photon-to-plasmon transducers (**c,d**) (oriented vertically as depicted by sketch). (**a**) EMCCD image of a test measurement showing two spots. The upper stems from the scattered excitation laser light at the first grating and the lower from out-coupled light from the ‘out-coupler’ grating. (**b**) Power P_out_ (and overall-efficiencies 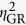) measured at the ‘out-coupler’ gratings of ten different test waveguide-structures (without plasmonic elements). The dashed vertical line and the shaded region show the mean value of 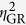 = 12.4 ± 1.7% and the uncertainty region of one standard-deviation, respectively. This mean value yields an efficiency of a single grating coupler of *η*_GR_ = 35 ± 2%. (**c**) CCD camera image corresponding to a measurement for estimating the coupling efficiency *η*_spp_ of the photon-to-plasmon transducer. The large upper spot stems from scattering of an expanded laser at the first Bragg-grating, the small spot (marked by a black circle) correspond to scattered out-coupled photons from the second Bragg-grating. The distance between two opposing grating couplers is approximately 50 *μ*m. (**d**) Measured relative out-coupled flux (crosses) as a function of plasmonic waveguide length L. The solid blue curve is a double exponential fit to the signal, showing that typical SPP damping is present. Dashed lines show the contributions of the fast and slowly decaying components, respectively. Fitting the slow decay at zero waveguide length yields a coupling efficiency of *η*_spp_ = (57 ± 21)%. The coupling efficiency seems to be high for short length L only due to direct coupling of the photonic waveguides by scattered photons (fitted by the fast exponential decay).

First we investigate the far-field grating couplers and then concentrate on the photon-to-plasmon transducer. The grating couplers have been adapted from refs 22 and 28. They consist of two gratings, where the coarse grating performs the actual coupling and the second, finer grating acts as a Bragg-mirror to enhance the directionality of the coupling, into the dielectric waveguide. For the measurements on the efficiency of the grating couplers, a focused beam coming from an objective with a numerical aperture (NA) of 0.4 is used. In order to derive the efficiency, the total irradiated laser power needs to be known. To this end, the reflection of the laser spot from a flat silver mirror (assumed to be almost 100% reflective) was used to determine the absolute incoming power *P*_tot_ (measured in counts on an Andor electron multiplying charge-coupled device (EMCCD) camera). During the analysis of the grating couplers, the reflection of the laser on a flat chip-area next to the waveguide structures (*P*_reflect_) was repeatedly measured to track the laser’s power stability. The actual in- and out-coupling to and from the waveguide structures was adjusted with micrometer screws, an x-y piezo stage and a piezo to align the focus until the out-coupling (*P*_out_) was optimized. As two grating couplers are used here that are connected via a waveguide, we actually measure the product of two efficiencies *η*_in_ ⋅ *η*_out_ ≈ 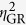 (where 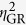 = *P*_out_/*P*_tot_), so that the root of the result gives the average coupling efficiency *η*_GR_ of a single grating coupler. The procedure was performed for a series of waveguide structures (Fig. 4a,b) and an efficiency of *η*_GR_ = 35 ± 2% was found.

Now we study the performance of the photon-to-plasmon transducers. In particular the coupling efficiency *η*_spp_ is determined, given by the ratio of the excited SPPs to the number of the incoming photons in the dielectric waveguide. To this end, we compare the transmission of the fabricated photonic-plasmonic structures to the transmission of purely dielectric reference structures fabricated on the same sample. Note that variations in transmission through the reference structures are below 2% due to the high quality of the fabrication process, ensuring the comparability with the results from Fig. 4a,b). In this series of measurements, we use an inverted microscope with a CCD camera for detection. One of the Bragg-gratings on the end of the dielectric waveguide is fully illuminated by a weakly focused laser (*λ* = 785 nm). The sample is aligned to optimize the transmission to the Bragg-grating at the opposite end. Images are taken by the CCD camera and analyzed (Fig. 4c). By normalizing the intensity at the output grating with respect to the flux emerging from the reference structures without plasmonic components, the overall efficiency of the plasmonic components is determined. To determine the propagation losses in the plasmonic waveguides and to extract the efficiency *η*_spp_ of the photon-to-plasmon transducers, the measurement is repeated for a series of waveguide structures with different plasmon-waveguide length L (Fig. 4c,d): A fitting with a double-exponential (f(z) = A*e*^−*αz*^ + B*e*^−*βz*^) is performed. The extrapolation of the slowly decaying component of this fit to L = 0 reveals a conversion efficiency 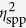 = (32 ± 12) % for two transducers and hence *η*_spp_ = (57 ± 21) % can be deduced for a single photon-to-plasmon transducer. This value is consistent with numerical simulations (analog to ref. 23) of the fabricated structures predicting *η*_spp,FEM_ = 40%. The exponential fit in Fig. 4d) matches the data points for large lengths L as it is expected for a plasmon waveguide with propagation losses leading to an exponential damping. The lower decay-constant corresponds to an attenuation constant *α* = (0.54 ± 0.14) *μm*^−1^ or a SPP propagation length of *λ*_spp_ = (1.84 ± 0.47) *μ*m, i.e., the distance at which the SPP’s intensity decays to 1/e of the initial intensity. This is in agreement with FEM simulations where *α*_FEM_ = 0.49 *μm*^−1^ and *λ*_spp,FEM_ = 2.05 *μ*m were found, respectively. For very short waveguide lengths the apparent coupling is larger than the estimated photon-to-plasmon conversion efficiency, since photons directly couple into the second dielectric waveguide by scattering. From the fast decay-constant, we can conclude, that the plasmon waveguide in this device needs to have a length of about L_min_ ≈ 2 *μ*m in order to avoid direct photon-coupling between the dielectric waveguides. Such direct coupling would manifest in a reduced signal-to-noise ratio of the envisioned effects as photons could pass the quantum emitter without interacting with it.

Based on this result, we conclude that our numerical approach (discussed in detail elsewhere^23^) can indeed give a quantitative estimate for the performance of the on-chip structures. Further we conclude that although the fabrication procedure is more complicated than for purely dielectric or purely plasmonic structures and although idealized conditions (ignoring roughness, defects, etc.) have been assumed within the computations, we can indeed reach the high efficiencies found in numerical simulations.

### Numerical optimizations for high Purcell and *β*-factors

In this section we estimate the non-linear performance of the device when coupled to emitters. It is essential to establish a high Purcell factor *P* and a high *β*-factor at the same time. This can be seen, e.g., by looking at the reflectance R of a single emitter onto a single impinging guided photon/SPP (according to Chang *et al*.^4^):


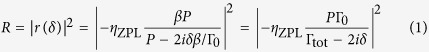


with the detuning between the emitter’s transition and the SPP’s frequency *δ*, the Debye-Waller factor *η*_ZPL_ and the unperturbed (vacuum) decay rate of the emitter Γ_0_. At resonance (*δ* = 0) this simplifies to R = (*η*_ZPL_ ⋅ *β*)^2^. In a concrete experiment, the transmittance T would drop when the incoming light, e.g., from an attenuated laser, is scanned over the emitter’s transition. A large magnitude of the Purcell factor *P* is relevant, as two features will be enhanced: (i) the width of the transmittance dip and (ii) the potential rate of impinging SPPs that the emitter can handle before it saturates, which would destroy its ability to reduce the transmittance. As mentioned earlier, *β* relates the decay rate Γ_WG_ induced by the guided mode to the overall decay rate Γ_tot_. In any plasmonic structure the latter will typically exceed Γ_WG_ by orders of magnitude when the corresponding emitter is very close to a metallic surface (see e.g.^26^,^29^,^30^) which is typically ascribed to electron-hole pair creation. As the field of the excited mode in the simple plasmonic stripe waveguide is closely localized to the metal, the hot spots coincide spatially mainly with areas of strong quenching (high Γ_tot_) leading to poor *β*-factors.

**Figure 5:**
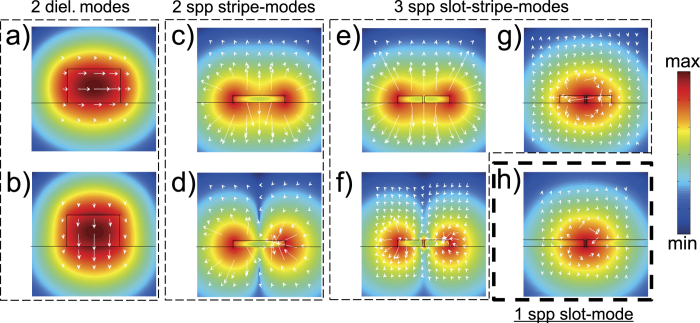
Waveguide modes. (**a–h**) Logarithmic intensity distributions and polarizations indicated by white arrows. (**a**) The TE mode in the dielectric waveguide, (**b**) the corresponding TM mode, (**c**) the fundamental and (**d**) 2^nd^ SPP mode of a simple stripe waveguide, (**e,f**) the corresponding modes of a slotted stripe waveguide, (**g**) the 3^rd^ mode for this cross-section, i.e., the slot-stripe mode and (**h**) the sole and highly localized plasmonic slot-mode featuring a polarization allowing for excitation by the TE mode of the dielectric waveguide. All subfigures have separate color-gradients.

**Figure 6:**
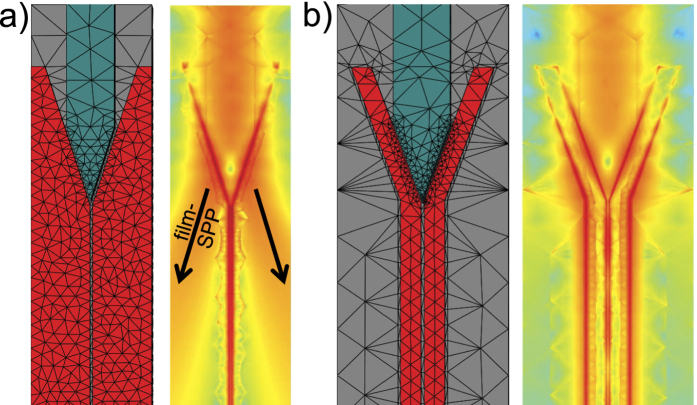
3D FEM meshs and simulated intensity plots of the electric field intensity (logarithmic scale). (**a**) The transducer with a simple slot-waveguide suffers from parasitic excitation of film modes (indicated by arrows). (**b**) Two modes excited in the slot-stripe waveguide, leading to a beating effect and - importantly - allowing SPPs to bypass emitters in the slot which undermines the performance of the device.

Thus, Chang *et al*.^4^ suggest to taper the plasmonic waveguide down to less than 50 nm to enter a regime where both *P* and *β* reach sufficiently high values for the fundamental guided mode. It turns out, that this approach of tapering the width of the on-chip waveguide fails for our transducer-design, as the transducer does not couple to the fundamental mode but almost entirely to the next higher mode which experiences a cut-off during tapering. The dielectric waveguides on the chip support almost purely transversal magnetic (TM) or transversal electric (TE) modes (Fig. 5a,b) with a nearly linear polarization perpendicular to the propagation direction. In contrast, the fundamental plasmonic mode, i.e., the only plasmonic mode, which does not suffer from a cut-off when tapered down, features a nearly radial polarization of a breathing mode (Fig. 5c). Consequently, the symmetries of the guided dielectric and the fundamental plasmonic mode do not fit to each other and a high coupling efficiency between these modes cannot be expected with our transducer design. It rather exclusively couples to the mode depicted in Fig. 5d). This problem can be tackled with a plasmonic slot-waveguide. These waveguides can be constructed simply by a slot in a metallic film (Fig. 5h) or by two parallel stripe waveguides, yielding a ‘slot-stripe’ waveguide (Fig. 5e–g). Specifically, a mode is supported, that channels most of the electromagnetic flux through the slot which promises high Purcell factors. Most importantly, the polarization matches better to the dielectric waveguide modes. For this reason we adapted our transducer design to work with a slot-waveguide. Figure 6 shows a simulation of an adapted transducer feeding a slot-waveguide in a metal film (similar to the transducer-design by Tian *et al*. but with a much narrower slot^31^) and a slot-stripe waveguide, respectively. On first glance the slot-stripe waveguide (Fig. 6b) promises much better coupling efficiency with respect to the ordinary slot-waveguide (Fig. 6a) where film SPPs are excited that represent a loss channel. However, the transmission through the slot-stripe waveguide is not based on a single mode. Actually, mainly a mode with hot-spots at the outer edges of the two stripes is excited, thus bypassing emitters inside the slot. In an optimization for the coupling efficiency to the simple slot-waveguide, we found an efficiency of *η*_slot_ = 11.2%. The efficiency can be significantly increased for wider slots^31^, thus a high efficiency together with a high Purcell factor can be achieved by tapering the slot-waveguide (which does not suffer from a cut-off).

### A realistic single photon transistor

We will now investigate the Purcell factor *P* and *β*-factor *β* for a straight (no tapering) slot-waveguide, as it was used in the simulations shown in Figs 5h and 6a. We chose a slot-waveguide cross-section with the same gold-thickness as for the fabricated structures and a slot width of 30 nm. This slot width is within the current fabrication accuracy. As strong quenching typically sets in at emitter-metal distances below 5 nm^29^,^30^, we expect regions of both high P’s and *β*’s centered in the slot. For the corresponding slot-mode (Fig. 5h) we find propagation losses of *α*_slot_ = 1.02 *μm*^−1^. To compute *P* we follow the method published by Barthez *et al*.^30^ using:


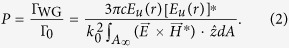


**Figure 7:**
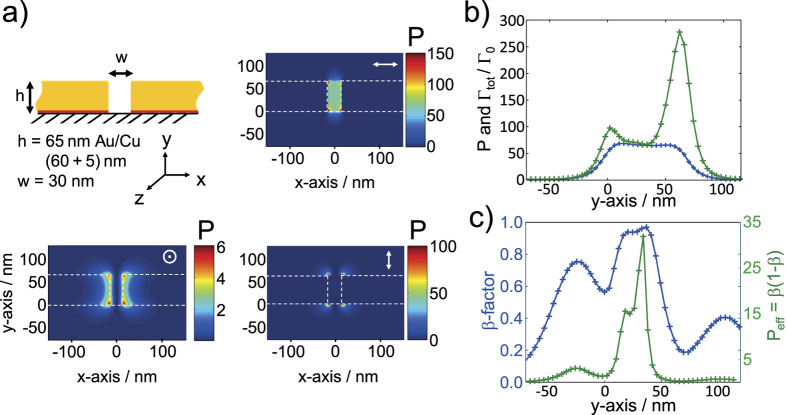
Coupling of emitters to the plasmonic slot mode. (**a**) Purcell factor *P* of the plasmonic slot-stripe waveguide for three linearly independent orientations of the emitters’ dipoles calculated with equation 2^30^. A dipole orientation along x features high *P*’s in the entire slot. (**b**) Purcell factor *P* (blue) and the normalized total decay rate Γ_tot_/Γ_0_ (green) for an emitter’s dipole oriented along x, centered in the slot and scanned along y. (**c**) Corresponding *β*-factor (blue) and - for comparison with Chang *et al*.^4^ - the ‘effective Purcell factor’ *P*_eff_ = *β*(1 − *β*) (green) also as a scan along y.

Here 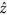 is a normalized vector pointing in direction of propagation along the waveguide which is normal to the surface *dA. k*_0_ is the absolute value of the photon momentum in air. *E*_*u*_ denotes the electric field components parallel to the dipole orientation of an emitter. This computation can be performed very quickly, as only the solution of a frequency-domain guided-mode solver (here JCMwave) is needed. The resulting map of *P* is shown in Fig. 7a) for three linearly independent dipole orientations. The computation of the *β*-factor is more complicated as Γ_tot_ is needed. A common method to derive Γ_tot_ is to let a dipolar point-source radiate in a full 3D calculation and to monitor its total emitted power with respect to the emitted power in vacuum. To avoid any back-action of the emitted power onto the dipole like additional resonant Purcell effects, we use here a straight slot-waveguide that is long (9 *μ*m) compared to the mean-propagation distance of 1/*α*_slot_ = 0.98 *μ*m to damp away all outgoing power. In principle for each emitter-position of interest a new computation is needed. Here we can significantly reduce the amount of computations as we are looking for the optimum performance. Specifically only a single emitter orientation promises high values of *P* and only positions in the very center of the slot are of interest. Any position closer to the gold will for sure enhance the quenching^26^,^29^,^30^ but only slightly affect *P*, as *P* is quite homogeneous in the slot (see Fig. 7a). The (normalized) total decay rate Γ_tot_/Γ_0_, *P* and *β* scanned along the center of the slot, are shown in Fig. 7b,c) yielding an optimum of *β* = 97% at *P* ≈ 64. Γ_0_ denotes the undisturbed decay rate of the emitter (in vacuum).

**Figure 8:**
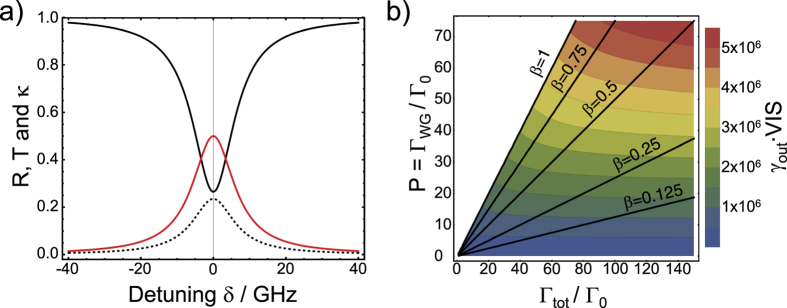
Performance of the single-photon transistor. (**a**) Reflectance R (dashed), transmittance T (solid black) and losses *κ* (solid red) as a function of detuning *δ* for the optimum values found for *P* and *β* as shown in Fig. 7b,c, however including a *η*_ZPL_ of 0.5. (**b**) *Performance γ*_out_ ⋅ VIS of the chip expected for a concrete experiment in which transmission dips are traced as a function of normalized total decay rate Γ_tot_/Γ_0_ and Purcell factor *P*. Every emitter position and orientation translates into a different position in this contour plot. The black lines mark specific *β*-factors. The white area refers to values of *β* > 1, which are not possible.

We can now quantify the *performance* of a non-linear quantum device. When we consider a single emitter in the slot waveguide controlling the transmittance T and reflectance R, then a figure of merit can be defined as the product of the collected photons from the chip *γ*_out_ and the visibility VIS of a transmission dip. This figure of merit corresponds perfectly to experimentally accessible quantities as was shown in ref. 5. In order to do this we insert values for *β* and *P* into equation 1 assuming a branching ratio for DBT of *η*_ZPL_ = 50%. With this we can directly plot T, R and the losses to the environment *κ (κ* = 1 − R − T) (Fig. 8a). We define the visibility VIS of the corresponding transmission dips by VIS = 1 − T, which is maximal at zero detuning (*δ* = 0):


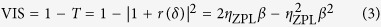


To derive the rate of outcoupled photons *γ*_out_ we first estimate the rate of impinging photons (SPPs) *γ*_in_, that are allowed without introducing saturation effects. To avoid saturation, two-photon events have to be avoided and the emitter has to be relaxed back to its ground state before the next excitation takes place. Now, we assume that the impinging photons or SPPs obey a Poisson distribution:


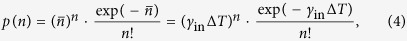


with the average photon number 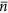 which is the rate of photons *γ*_in_ multiplied with time window Δ*T*. The restriction to have less than 1% chance of two-photon events and less than 1% chance to meet the emitter in its excited state can be formulated with the following relation:


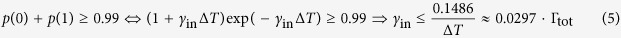


using Δ*T* = 5 ⋅ *τ* = 5/Γ_tot_. The restriction for Δ*T* is found by integrating the exponentially decaying chance to observe a decay process which yields 99.3% after 5 lifetimes *τ*. Please note, that if the attenuated laser would be replaced by a perfect single photon source the allowed rate of incoming photons could be roughly 10 times higher, to be specific *γ*_in_ would rise to the aforementioned value of Γ_tot_/5.

To derive the number of photons that can be collected from the chip, the imperfect efficiency of the chip has to be considered, yielding:


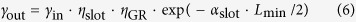


where we use the nominal transducer and grating coupler efficiencies, and assume that the emitter is placed half-way next to the plasmonic waveguide of length *L*_min_ = 2 *μ*m. With an approximate excited state lifetime of DBT *τ*_0_ = 5 ns (Γ_0_ = 1/*τ*_0_ = 2 ⋅ 10^8^ *s*^−1^)^20^,^21^,^32^ in AC we can now plot the expected performance (*γ*_out_ ⋅ VIS) as a function of Purcell factor *P* and normalized total decay rate (Fig. 8). For an optimum emitter orientation and position in the center of the slot we expect a value of *γ*_out_ ⋅ VIS ≈ 4.07 ⋅ 10^6^ photons per second.

## Discussion

To summarize, we described the fabrication of photonic-plasmonic on-chip hybrid structures. The performance of the structures was analyzed, finding excellent agreement with numeric simulations^23^. This is an essential step, as we can conclude to have the numerical tools to quantitatively predict the performance of the actual devices. We compute actual parameters for the non-linear behavior of a single photon transistor consisting of a plasmonic-dielectric slot-waveguide structure coupled to organic DBT emitters. These concrete parameters show that observable non-linearities at the single-photon and single-emitter level can indeed be expected with this design. Further, the adapted design utilizing a plasmonic slot-waveguide holds the potential for (DC-)Stark-shifting the molecules’ transition-frequency by simply contacting the gold film on either of the slot’s sides and applying a voltage. Besides organic emitters like DBT, the on-chip structures could in principle also be coupled to cooled Rubidium-atoms as suggested and demonstrated for simple gold-films by Stehle *et al*.^33^. The D2-line of Rubidium is found at 780 nm and thus matches to the current working frequency. We are convinced that our work helps to pave the way towards the next level of integrated quantum plasmonic experiments.

## Additional Information

**How to cite this article**: Kewes, G. *et al*. A realistic fabrication and design concept for quantum gates based on single emitters integrated in plasmonic-dielectric waveguide structures. *Sci. Rep.* **6**, 28877; doi: 10.1038/srep28877 (2016).
